# An Attempt at Captive Breeding of the Endangered Newt *Echinotriton andersoni*, from the Central Ryukyus in Japan

**DOI:** 10.3390/ani3030680

**Published:** 2013-07-31

**Authors:** Takeshi Igawa, Hirotaka Sugawara, Miyuki Tado, Takuma Nishitani, Atsushi Kurabayashi, Mohammed Mafizul Islam, Shohei Oumi, Seiki Katsuren, Tamotsu Fujii, Masayuki Sumida

**Affiliations:** 1 Institute for Amphibian Biology, Graduate School of Science, Hiroshima University, 1-3-1 Kagamiyama, Higashihiroshima 739-8526, Japan; E-Mails: tigawa@hiroshima-u.ac.jp (T.I.); sugawara-hirotaka@ed.tmu.ac.jp (H.S.); m131250@hiroshima-u.ac.jp (M.T.); nitroof1@hotmail.com (T.N.); kuraba@hiroshima-u.ac.jp (A.K.); mafizul77@yahoo.com (M.M.I.); 2 Section of Agriculture and Forestry, Amami City Government, Amami, Kagoshima 894-0048, Japan; E-Mail: shoooumi@city.amami.lg.jp; 3 Okinawa Prefectural Institute of Health and Environment, Okinawa 901-1202, Japan; E-Mail: skatsuren@gmail.com; 4 Faculty of Human Culture & Science, Hiroshima Prefectural University, Hiroshima 734-8558, Japan; E-Mail: fujii@pu-hiroshima.ac.jp

**Keywords:** captive breeding, endangered newt, natural monument, IUCN Red List, *Echinotriton andersoni*

## Abstract

**Simple Summary:**

We naturally bred the endangered Anderson’s crocodile newt (*Echinotriton andersoni*) and tested a laboratory farming technique using near-biotopic breeding cages with several male and female pairs collected from Okinawa, Amami, and Tokunoshima Islands. This is the first published report of successfully propagating an endangered species by using breeding cages in a laboratory setting for captive breeding. Our findings on the natural breeding and raising of larvae and adults are useful in breeding this endangered species, and can be applied to the preservation of other similarly wild and endangered species.

**Abstract:**

Anderson’s crocodile newt (*Echinotriton andersoni*) is distributed in the Central Ryukyu Islands of southern Japan, but environmental degradation and illegal collection over the last several decades have devastated the local populations. It has therefore been listed as a class B1 endangered species in the IUCN Red List, indicating that it is at high risk of extinction in the wild. The species is also protected by law in both Okinawa and Kagoshima prefectures. An artificial insemination technique using hormonal injections could not be applied to the breeding of this species in the laboratory. In this study we naturally bred the species, and tested a laboratory farming technique using several male and female *E. andersoni* pairs collected from Okinawa, Amami, and Tokunoshima Islands and subsequently maintained in near-biotopic breeding cages. Among 378 eggs derived from 17 females, 319 (84.4%) became normal tailbud embryos, 274 (72.5%) hatched normally, 213 (56.3%) metamorphosed normally, and 141 (37.3%) became normal two-month-old newts; in addition, 77 one- to three-year-old Tokunoshima newts and 32 Amami larvae are currently still growing normally. Over the last five breeding seasons, eggs were laid in-cage on slopes near the waterfront. Larvae were raised in nets maintained in a temperature-controlled water bath at 20 °C and fed live *Tubifex*. Metamorphosed newts were transferred to plastic containers containing wet sponges kept in a temperature-controlled incubator at 22.5 °C and fed a cricket diet to promote healthy growth. This is the first published report of successfully propagating an endangered species by using breeding cages in a laboratory setting for captive breeding. Our findings on the natural breeding and raising of larvae and adults are useful in breeding this endangered species and can be applied to the preservation of other similarly wild and endangered species such as *E. chinhaiensis.*

## 1. Introduction

**Figure 1:**
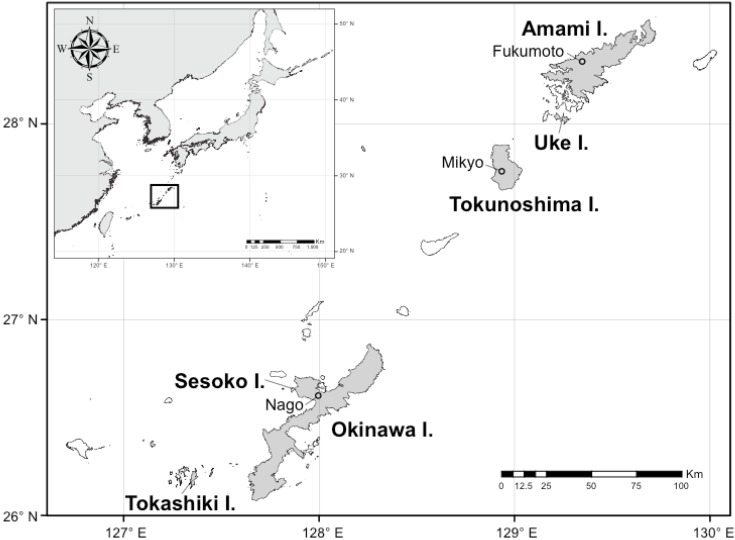
Map showing the distribution of *Echinotriton andersoni* in the Ryukyu Islands, and the collection sites.

Anderson’s crocodile newt, *Echinotriton andersoni*, a species endemic to the Ryukyu Islands of southwest Japan (Figure 1), has been described as both a primitive newt and a living fossil originating from the Cenozoic Tertiary period, and is distributed in six islands of the Central Ryukyus—namely, Amami, Uke, Tokunoshima, Okinawa, Sesoko, and Tokashiki Islands [1,2]. This species was also formerly observed in Taiwan [3], where it is currently presumed to be extinct [4], although this requires careful verification [5]. Regrettably, the species has been devastated over the last several decades by illegal collection for the pet trade, predation by the Java mongoose, an invasive species known for its negative impact on native wildlife in Japan [6], and environmental degradation. *E. andersoni* is now listed as a class B1 endangered species [7] and is designated as a natural monument in both Okinawa and Kagoshima prefectures [8]. It is urgently necessary, therefore, to protect local populations where the number of individuals has decreased or environmental conditions have worsened to levels such that the species is unable to survive by itself and is critically endangered.

The natural breeding habitats and behaviors of *E. andersoni* have been observed in detail both in the wild [9,10] and in the laboratory [11,12,13,14]. In a study by Utsunomiya and Matsui [11], a male crept around a female, sniffing around her body and drawing a thread of mucus from his cloacae so that the female was surrounded by spider web-like strings of mucous attached to the substrate. The male then deposited a spermatophore by rubbing his cloacae against the substratum while swaying his body back and forth. 

It has been reported that several frog species show genetic and morphological variations between Okinawa and the Amami Islands, and they have even recently been described as different species [15,16,17]. Allozyme and mtDNA analyses have been conducted with the purpose of clarifying genetic diversity among wild populations of *E. andersoni* [18,19,20]. Microsatellite analysis is a focus of current study by Sugawara *et al.* [21] and [22] (Igawa *et al.*, in preparation). Based on these studies, *E. andersoni* was found to diverge into several local groups. 

In the present study, we performed captive breeding of this endangered newt, evaluated a rearing technique, and promoted natural breeding in a laboratory setting in an attempt to aid efforts to preserve this endangered species and so conserve the genetic diversity of wild populations.

## 2. Materials and Methods

**Figure 2:**
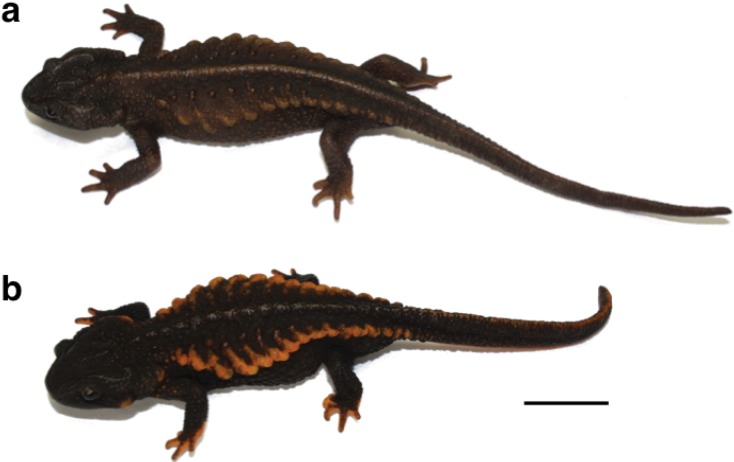
Representative newts from two populations of *E. andersoni*. (**a**) Okinawa population. (**b**) Tokunoshima population. Scale bar = 2.0 cm.

**Figure 3:**
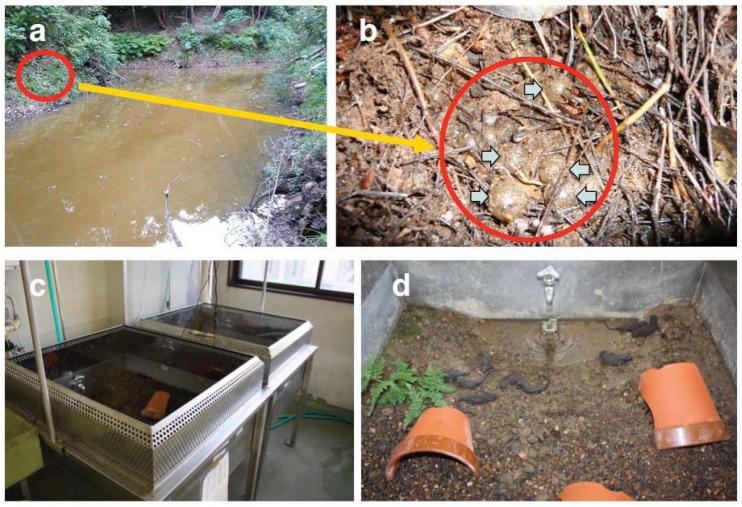
(**a**, **b**) Outdoor breeding site at Nago, Okinawa Island. Arrows indicate deposited eggs. (**c**, **d**) Breeding cages (90 × 90 × 50 cm) manufactured to be near-biotopes.

**Figure 4:**
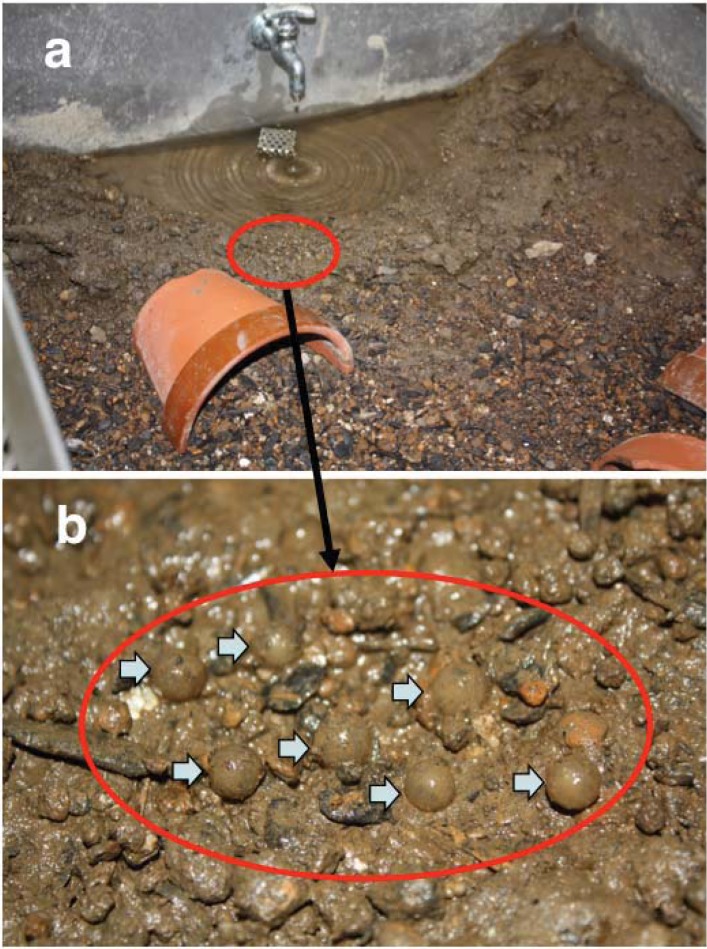
Eggs deposited on the slope in the vicinity of the waterfront in the cage. (**a**) The slope in the vicinity of the waterfront in the breeding cage, not directly submerged. (**b**) Eggs deposited on the slope are indicated by arrows.

**Figure 5:**
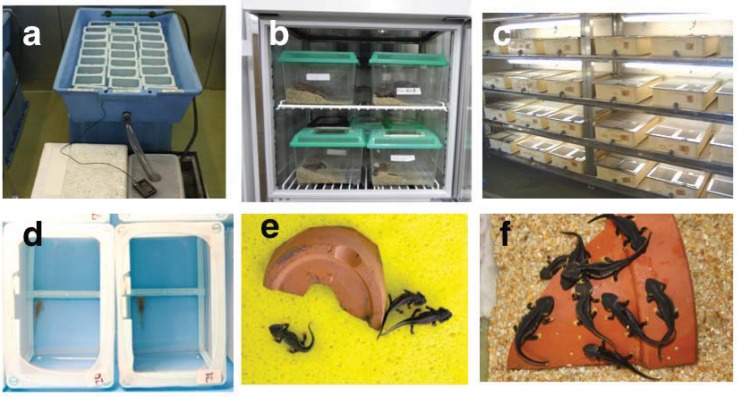
(**a**, **b**) Larvae were raised in nets maintained in a temperature-controlled water bath at 20 °C and fed live *Tubifex*. (**b**, **e**) Metamorphosed newts were transferred to plastic containers containing wet sponges, kept in a temperature-controlled incubator at 22.5 °C, and fed a diet of crickets. (**c**, **f**) One-year-old newts were housed in our frog room controlled at 25 °C and fed a diet of crickets.

We used three populations (from Okinawa, Amami, and Tokunoshima Islands) of *E. andersoni* for captive breeding in the laboratory (Figure 1). Live animals were collected with permission from the Boards of Education of both Kagoshima and Okinawa prefectures: 11 females and two males from Nago, Okinawa Island, five females and 11 males from Mikyo, Tokunoshima Island, and two females and four males from Fukumoto, Amami Island (Figure 2). Specimens were fed earthworms and maintained separately with respect to each island population in near-biotopic breeding cages of the same sizes (90 × 90 × 50 cm) (Figure 3(c)). The breeding cages were set up to imitate the wild breeding site at Nago, Okinawa Island (Figure 3(a,b)). A depressed area in the shape of an inverted half-cone (300 mm diameter, 200 mm depth, and 15° slope angle) attached to a water drainage pipe (100 mm from the bottom) was made on one side of each cage and filled with water (Figure 3(d)). A water faucet was attached above the sink and tap water was poured at approximately 150 mL per hour. A 70:30 v/v mixture of leaves and paddy soil was used as floor cover, except in the depressed area and in the vicinity of areas coated only by paddy soil. Water depth in the depressed area was regulated to 300 mm by depositing paddy soil. A constant condition was maintained at 18–25 °C with 60–80% humidity and 10 h of lighting from 8 a.m. to 6 p.m. Although we did not give any stimulus to induce reproductive behavior, captive breeding was performed naturally by male and female *E. andersoni* pairs in each cage. Eggs were found deposited on the slopes in the vicinity of the cages’ waterfronts, but not directly submerged, during the breeding seasons (Figure 4). Larvae were raised in nets maintained in a temperature-controlled water bath at 20 °C and were fed live *Tubifex* (Figure 5(a,b)). Metamorphosed newts were transferred to plastic containers containing wet sponges, kept in a temperature-controlled incubator at 22.5 °C, and fed a diet of crickets (Figure 5(b,e)). One-year-old newts were housed in our frog room controlled at 25 °C and fed a diet of crickets (Figure 5(c,f)). At each stage, the normally developed embryos and larvae were counted, and developmental capacity (survival rate) was calculated.

## 3. Results

### 3.1. Development of Naturally Deposited Embryos

**Figure 6:**
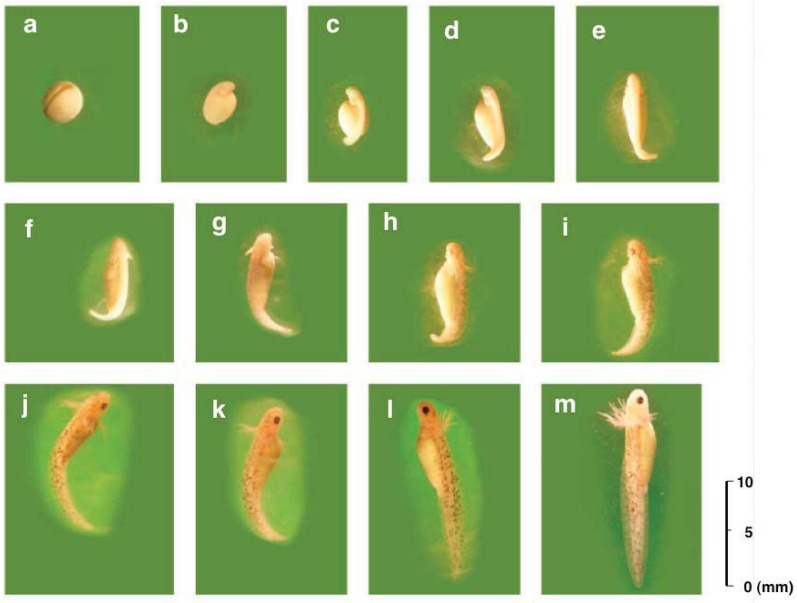
Normal development from the neurula to hatching stages: (**a**) 8-day-old neurula embryo, (**b**) 9-day-old tailbud embryo, (**c**) 10-day-old larva, (**d**) 11-day-old larva, (**e**) 12-day-old larva, (**f**) 13-day-old larva, (**g**) 14-day-old larva, (**h**) 15-day-old larva, (**i**) 16-day-old larva, (**j**) 17-day-old larva, (**k**) 18-day-old larva, (**l**) 19-day-old larva, (**m**) 20-day-old hatched larva.

Eggs were deposited on the slope in the vicinity of each cage’s waterfront during the breeding seasons over the 5 years of the breeding program (Figure 4). Normal development was shown from the neurula to hatching stages (Figure 6). Eggs of this species were greyish and comparatively large, with a diameter of 3.0 ± 0.1 mm. Most embryos reached the neurula stage eight days after oviposition (Figure 6(a)), the tailbud stage nine days after oviposition (Figure 6(b)), and hatched into larvae at 20 days after oviposition (Figure 6(m)). Finally, they completed metamorphosis within 3–4 months of oviposition. The present data were compared with those of Utsunomiya and Utsunomiya [23] and no differences were found.

### 3.2. Developmental Capacity

**Figure 7:**
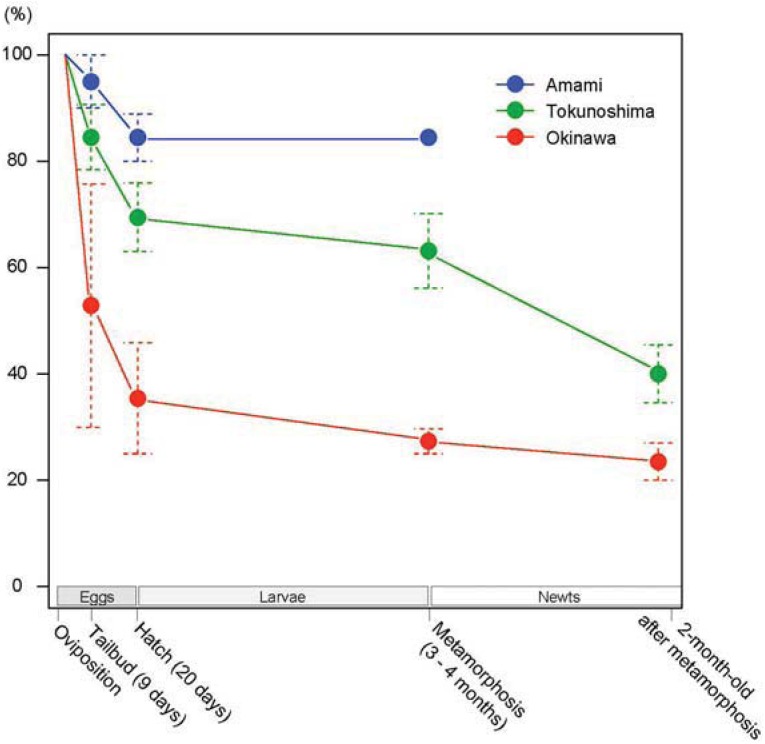
Survival curves showing developmental capacity of the offspring of the Okinawa, Amami, and Tokunoshima Island populations produced through captive breeding in cages in the laboratory setting. Beginning with the deposited eggs, viability at four developmental stages was monitored to evaluate developmental capacity. The vertical bars show the standard error at each stage.

**Figure 8:**
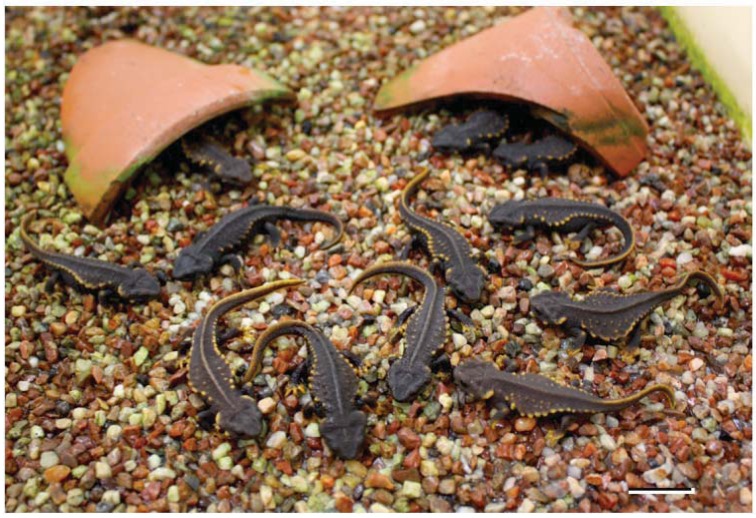
Three-year-old newts (body length 8–9 cm) produced by captive breeding and raised in the laboratory. Scale bar = 2.0 cm.

Developmental capacity of the embryos produced by oviposition is shown in Table 1. Survival curves showing developmental capacity of the embryos are shown in Figure 7. Both intra- and inter-island developmental capacity was different. Among the 378 eggs derived from 17 females, 319 (84.4%) became normal tailbud embryos, 274 (72.5%) hatched normally, 213 (56.3%) metamorphosed normally, and 141 (37.3%) became normal 2-month-old newts; 32 Amami larvae are currently still growing normally. A comparatively high mortality was found between the tailbud and hatching stages, and around metamorphosis. Roughly 20–70% of deposited eggs constantly achieved metamorphosis across the 5 breeding seasons, although all eggs died due to desiccation before hatching in the wild [8]. At present, 26 one-, 5 two-, and 46 three-year-old Tokunoshima newts produced by the natural breeding program are being raised in our laboratory (Figure 8).

**Table 1:** Developmental capacity of offspring produced by captive breeding.

Island	Date	No. of Eggs	Normal Tailbud Embryos (%)	Normally Hatched Larvae (%)	Metamor- phosed Newts (%)	2-Month-Old Newts (%)
**Okinawa**	2009.4.19	37	28 (75.7)	17 (45.9)	11 (29.7)	10 (27.0)
	2010.4.10	20	6 (30.0)	5 (25.0)	5 (25.0)	4 (20.0)
**Total**		57	34 (59.6)	22 (38.6)	16 (28.1)	14 (24.6)
**Tokunoshima**	2010.2.22	33	29 (87.9)	29 (87.9)	29 (87.9)	22 (66.7)
	2010.3.23	21	21 (100)	21 (100)	21 (100)	16 (76.2)
	2010.4.12	30	25 (83.3)	24 (80.0)	23 (76.7)	13 (43.3)
	2010.4.16	17	9 (52.9)	9 (52.9)	9 (52.9)	4 (23.5)
	2010.6. 4	36	25 (69.4)	25 (69.4)	25 (69.4)	14 (38.9)
	2011.1.23	44	37 (61.4)	37 (61.4)	25 (56.8)	14 (31.8)
	2011.3.11	20	20 (100)	10 (50.0)	10 (50.0)	6 (30.0)
	2011.4.13	3	3 (100)	3 (100)	3 (100)	2 (66.7)
	2011.4.30	10	10 (100)	5 (50.0)	4 (40.0)	2 (20.0)
	2011.5. 7	17	17 (100)	7 (41.2)	4 (23.5)	2 (11.8)
	2011.5.29	7	7 (100)	6 (85.7)	6 (85.7)	4 (57.1)
	2012.3. 7	46	46 (100)	44 (95.7)	38 (82.6)	28 (60.9)
**Total**		284	249 (87.7)	220 (77.5)	197 (69.4)	127 (44.7)
**Amami**	2013.4.17	27	27(100)	24(88.9)	24(88.9)	–
	2013.5.21	10	9(90.0)	8(80.0)	–	–
**Total**		37	36 (97.3)	32 (865)	–	–

–: Currently developing.

## 4. Discussion

The artificial insemination technique described by Nishioka [24], which involves the injection of a bullfrog pituitary hormone into the female body cavity to induce ovulation, has been applied to numerous species. We previously demonstrated that this technique could also be applied to the artificial breeding setup of *Odorrana ishikawae*, also listed as a class B1 endangered species in the IUCN Red List and designated as a natural monument in both Okinawa and Kagoshima prefectures. Sumida *et al.* [25] tested a farming technique involving an artificial breeding setup for frogs and also promoted natural breeding in the laboratory in an effort to help preserve the endangered species. After rearing these artificially bred frogs in the laboratory, second-generation frogs were successfully produced through natural mating activities. A similar artificial insemination technique was used with *E. andersoni* by Utsunomiya and Utsunomiya [23], which involved the injection of a newt pituitary hormone into the female body cavity to induce ovulation. However, in our study, we found that this technique cannot be applied to the breeding of *E. andersoni* in the laboratory as it scarifies males for artificial insemination. Thus, we instead attempted captive breeding in the laboratory using the breeding cages created in this study. 

In the wild, *E. andersoni* is known to oviposit on slopes near waterfronts but not directly in the water [8]. The present study confirmed that when an analogous environment is created within a breeding cage, oviposition also occurs in and around waterfronts that are not directly submerged. Accordingly, successful oviposition is considerably more achievable by establishing this type of environment within a breeding cage, with a setting closely resembling natural conditions. Going forward, we hope that the use of this artificial breeding setup will ensure stable levels of oviposition. The ovipositing period for this species in its natural habitat is from February to June [26]. Our research revealed that oviposition in the artificial breeding setup occurred from the late February to early June. As there is no notable difference between the ovipositing period in nature and that in captivity, these periods are considered to be essentially the same.

To evaluate the feasibility of this captive breeding approach, the developmental capacity of naturally bred embryos was monitored in detail. Overall, embryo survivability during early development was not high, with fewer than 50% of deposited eggs achieving metamorphosis. In particular, the mortality rate was highest at the hatching and metamorphosis stages. On the other hand, Orton and Routledge [27] found low hatching failures and larval mortality in the common toad (*Bufo bufo*; an anuran that deposit its eggs in water), but high mortality at these stages when eggs and tadpoles were caged within their breeding sites (*i.e.*, the natural environment). Conversely, metamorph mortality was high in both environments.

One reason for the high mortality between the tailbud and hatching stages was the drying of the eggs deposited on land. The present study also found that the survival rate from oviposition to the tailbud stage is higher for eggs reared on waterside land when compared with those reared underwater (data not shown). Based on this finding, the survival rate until at least the tailbud stage is considered to be high for eggs reared on waterside land. In several cases, however, the embryos reared on such land died before hatching could occur (data not shown). When these findings are considered together, they indicate that survival rate can be increased by using the lowest amount of water possible, in order to prevent embryo desiccation until the tailbud stage, and thereafter, by gradually increasing the water until the hatching stage. 

Another reason for the high mortality between the hatching and metamorphosis stages was intraspecific predation. During the larval period, cannibalism was confirmed, even though there were only three individuals per container. To avoid cannibalism, a maximum of two individuals should be reared in each container, and ideally one individual. Another reason for the high mortality around the metamorphosis stage was a skin disease caused by a type of mold infection. The mortality rate from this skin disease before and after metamorphosis was high, although complete recovery was confirmed in a number of larvae that received treatment with methylene blue solution (1.4 mg/L) for 10 min. Early treatment of larvae during this period using methylene blue solution should lower the mortality rate.

The findings presented here regarding captive breeding and the raising of larvae are useful for the breeding of *E. andersoni* in the laboratory setting, and are applicable to the preservation of wild populations of this species. During the 2010 and 2011 breeding seasons, first-generation offspring were produced through the natural mating activities of male and female pairs kept in the breeding cages. This is the first published report of successful natural breeding in the laboratory. Thus, this technique can also be used for the temporary protection of local populations where the number of individuals has decreased or environmental conditions have worsened to levels that prevent the species from being able to survive without intervention. However, despite these promising results, it is necessary to evaluate the genetic diversity of naturally bred individuals and consider the impact of releasing these newts to surrounding ecosystems. 

As *E. andersoni* is geographically isolated between Amami, Okinawa, and Tokunoshima Islands, it is currently believed these populations are a result of the Ancient Island Split (about 3.2–1.5 Ma) [28,29,30]. With respect to these populations, geographic differences have been observed by allozyme analysis [18], mitochondrial cytochrome *b* gene sequences [20], complete mtDNA analysis [19], and microsatellite analysis [21,22]. Thus, it is important and necessary to conserve not only the Amami and Tokunoshima but also the Okinawa Island populations as endangered newt populations. In this study, we succeeded in sustainable captive breeding of individuals collected from these three locations for a series of years and could ultimately preserve the populations for newt diversity conservation.

## 5. Conclusions

In this study we naturally bred the endangered Anderson’s crocodile newt (*E. andersoni)* and tested a laboratory farming technique to preserve this species, using several male and female pairs from Okinawa, Amami, and Tokunoshima Islands that were maintained in near-biotopic breeding cages. Eggs were laid consistently in the breeding cages near the waterfront. The appropriate water temperatures for embryos and larvae are 20 °C and 22.5 °C, respectively. Larvae were fed live *Tubifex*, and metamorphosed newts were fed a diet of crickets, to encourage healthy growth. This is the first published report of the successful captive breeding of an endangered species in the laboratory using breeding cages. Our findings regarding the captive breeding and raising of larvae and adults may be useful for the breeding of endangered species and can be applied to the preservation of other similar wild endangered species in the future, although the present investigation can be viewed as a case study.
